# Activity of Protein Kinase A in the Frontal Cortex in Schizophrenia

**DOI:** 10.3390/brainsci14010013

**Published:** 2023-12-22

**Authors:** Smita Sahay, Nicholas Daniel Henkel, Christina Flora-Anabelle Vargas, Robert Erne McCullumsmith, Sinead Marie O’Donovan

**Affiliations:** 1 Department of Neurosciences, University of Toledo College of Medicine and Life Sciences, Toledo, OH 43614, USA; smita.sahay@rockets.utoledo.edu (S.S.); nicholas.henkel@rockets.utoledo.edu (N.D.H.); christina.vargas@rockets.utoledo.edu (C.F.-A.V.); robert.mccullumsmith@utoledo.edu (R.E.M.); 2 Neuroscience Institute, Promedica, Toledo, OH 43606, USA

**Keywords:** protein kinase A, enzyme activity, postmortem, frontal cortex, schizophrenia

## Abstract

Schizophrenia is a serious cognitive disorder characterized by disruptions in neurotransmission, a process requiring the coordination of multiple kinase-mediated signaling events. Evidence suggests that the observed deficits in schizophrenia may be due to imbalances in kinase activity that propagate through an intracellular signaling network. Specifically, 3′-5′-cyclic adenosine monophosphate (cAMP)-associated signaling pathways are coupled to the activation of neurotransmitter receptors and modulate cellular functions through the activation of protein kinase A (PKA), an enzyme whose function is altered in the frontal cortex in schizophrenia. In this study, we measured the activity of PKA in human postmortem anterior cingulate cortex (ACC) and dorsolateral prefrontal cortex (DLPFC) tissue from schizophrenia and age- and sex-matched control subjects. No significant differences in PKA activity were observed in male and female individuals in either brain region; however, correlation analyses indicated that PKA activity in the ACC may be influenced by tissue pH in all subjects and by age and tissue pH in females. Our data provide novel insights into the function of PKA in the ACC and DLPFC in schizophrenia.

## 1. Introduction

Schizophrenia is a complex and heterogeneous neuropsychiatric disorder associated with the dysregulation of multiple brain neurotransmitter systems [1,2,3] that are functionally associated with protein kinases. For example, 3′-5′-cyclic adenosine monophosphate (cAMP)-dependent pathways are coupled to the activation of neurotransmitter receptors through the enzyme protein kinase A (PKA) [4,5,6]. PKA is a serine/threonine kinase that, once activated by the second messenger cAMP, phosphorylates downstream target molecules to ultimately modulate vital cellular responses [7]. Alterations in the mRNA expression, protein abundance, and/or protein activity of PKA have been reported in the frontal cortex in neuropsychiatric disorders including suicide [8,9], major depressive disorder [9,10], bipolar disorder [11,12], autism [13], and Alzheimer’s disease [14]. While direct alterations in PKA mRNA or protein levels in schizophrenia have not been assessed, rodent studies have reported the differential effects of antipsychotics on PKA mRNA, protein, and activity levels in the cortex, hippocampus, and striatum [15], suggesting PKA abnormalities and the ability of antipsychotics to regulate PKA-mediated pathways. Antipsychotic treatment has also increased mRNA and protein levels of the gamma-aminobutyric acid A (GABA_A_) receptor and cAMP-responsive element-binding protein 1 (CREB1) in the rat nucleus accumbens [16], both of which are implicated in schizophrenia pathology and are regulated via PKA. In essence, direct changes in PKA have been reported in several neuropsychiatric illnesses, but less is known about this essential signal transducer in the frontal cortex in schizophrenia.

Glutamate and dopamine system dysfunction is hypothesized to underlie the pathophysiology of schizophrenia [17,18,19]. PKA is a key component of the signaling cascade downstream of dopamine receptors and its activation leads to the regulation of dopamine-response neurons and associated functions, such as reward and motivation [5,20]. In glutamatergic neurons, PKA regulates the function of N-methyl-D-aspartate (NMDA) and α-amino-3-hydroxy-5-methyl-4-isoxazolepropinonic acid (AMPA) receptors, both of which are critical for synaptic plasticity and learning [21,22]. For example, PKA signaling increases AMPA receptor cell surface expression and trafficking in an activity-dependent manner in cortical neurons [23]. Moreover, dysregulated AMPA receptor expression and localization is reported in the frontal cortex in schizophrenia [24,25,26], a process that is correlated with altered synaptic plasticity and brain dysconnectivity: core phenomena observed in this disorder [23,27]. Additionally, PKA signaling induces transcription factor 4 (TCF4), a protein associated with cognitive deficits in schizophrenia, in cortical neurons [28]. Deficits in PKA signaling are also reported in BCL11B-deficient medium spiny neurons (MSNs) derived from human pluripotent stem cells [29]. BCL11B is a transcription factor and schizophrenia-associated risk gene that regulates PKA signaling in MSNs. These neurons are key components of the striatum and cortico-striatal circuits that play a central role in regulating dopamine transmission, cognitive processes, and motor functions, all of which are disrupted in schizophrenia [30]. While significant changes in PKA and cAMP-associated pathways have been reported in vitro, the activity of PKA in the human brain in schizophrenia is not well characterized and remains a significant gap in our knowledge of the molecular and cellular mechanisms underlying the glutamatergic and dopaminergic abnormalities in this disorder.

Previous in silico work in our laboratory examined altered serine/threonine kinase activity in chronic schizophrenia utilizing human postmortem brain tissue from the anterior cingulate cortex (ACC) and found increased PKA levels in samples from schizophrenia subjects compared to control subjects [31,32]. Since this finding did not address whether PKA was functionally active in schizophrenia subjects, we were interested in understanding the following question: is the activity of PKA increased in schizophrenia subjects in frontal cortical brain regions? In the present study, we hypothesized that PKA activity may be significantly increased in the ACC and the dorsolateral prefrontal cortex (DLPFC), brain regions that are commonly both structurally and functionally affected in schizophrenia [33,34]. We tested our hypothesis by measuring the activity of PKA in the ACC and DLPFC in samples from a well-characterized collection of postmortem brains from subjects with schizophrenia and sex- and age-matched non-psychiatrically ill control subjects. 

## 2. Materials and Methods

### 2.1. Tissue Acquisition and Preparation

ACC (Brodmann area 24/32) postmortem brain tissue was obtained from the Mount Sinai NIH Brain and Tissue Repository (New York City, NY, USA). The cohort that was assayed consisted of schizophrenia (*n* = 20) and non-psychiatrically ill control (*n* = 19) subjects. DLPFC (Brodmann area 9) postmortem brain tissue was obtained from the Maryland Brain Collection and was distributed by the Maryland and Alabama Brain Collections. The cohort that was assayed consisted of schizophrenia (*n* = 15) and non-psychiatrically ill control (*n* = 18) subjects. Cohorts of approximately 15–20 subjects are standard in the field for postmortem brain studies [35,36,37,38,39]. The rationale for the choice of these brain regions is provided in the supplement (References [40,41,42,43,44,45,46,47] are cited in the Supplementary Material). All tissue was acquired with consent from next of kin with Institutional Review Board approval protocols from the respective brain repositories. All subjects were diagnosed with schizophrenia based on DSM-IV criteria. The subjects’ medical records were examined using a blinded medical chart review instrument, as well as in-person interviews with each subject and/or their primary caregiver, as previously described [48]. Medication status was deemed to be “on” if subjects were on antipsychotic medication in the last six weeks of life. All subjects were matched for sex, age, pH, and postmortem interval (PMI) in the ACC and the DLPFC (Table 1). Supplementary Tables S1 and S2 provide the demographics for all subjects in the ACC and DLPFC cohorts, respectively.

### 2.2. PKA Assay Optimization and Activity Measurements

PKA kinase activity was measured in ACC and DLPFC tissue homogenates using the commercially available colorimetric PKA kinase activity assay kit (Abcam, Cambridge, United Kingdom; Cat#: ab139435), adapted to postmortem brain. 

Protein concentration optimization: Serial dilutions (0.5 − 5.2 × 10^−5^ μg/μL) of protein sample were performed to ensure linear readings and determine the appropriate concentration (μg/μL) and amount (μg) of protein for the assay. Samples and 95 °C heat inactivated samples were run in triplicate and read at the recommended wavelength (450 nm) using the BioTek Cytation^TM^ 5 (Supplementary Table S3). 

Inhibitor concentration optimization: The concentration of the potent H-89 PKA inhibitor (IC_50_ = 50 nM) was optimized to demonstrate the specificity of our assay (Abcam, Cat#: ab143787). Briefly, 5 mg of H-89 was reconstituted in 385 μL of water and diluted 1:5 again to allow for full dissolution. A final working concentration of 5 mM was achieved. A 10-fold serial dilution was performed to determine the optimal concentration (mM) of H-89 to use for the assay. Each H-89 concentration was tested in duplicate and read at the recommended wavelength (450 nm) using the BioTek Cytation^TM^ 5 (Supplementary Table S4). 

Sample preparation and bicinchoninic acid (BCA) assay: To determine the amount of protein present in each sample for our PKA activity assay, we performed a BCA assay, according to the manufacturer’s protocol (ThermoFisher Scientific, Cat#: 23227), with the following specifications. Five 14 μm sections per subject were scraped from glass slides and resuspended in approximately 100–150 μL of MPER protein extraction reagent (ThermoFisher Scientific, Cat#: 78501) + 1X HALT protease and phosphate inhibitor (ThermoFisher Scientific, Cat#: 78429). Protein samples were diluted 1:10 in the same reagent mixture prior to being loaded onto the plate. The standard curve was prepared according to the manufacturer’s guide. The assay was incubated for 30 min at 60 °C. Samples and BCA standards were run in duplicate and read at the recommended wavelength (562 nm) using the BioTek Cytation^TM^ 5.

PKA activity assay in ACC and DLPFC tissue homogenates: A total of 1.875 μg of protein sample was assayed for PKA kinase activity according to the manufacturer’s protocol (Abcam, Cat#: ab139435) with the following changes. For the kinase reaction, the samples were incubated at 30 °C for 35 min. For the incubation step, the samples were incubated at room temperature for 25 min. To serve as a positive control and normalize our data, a pool of all samples was prepared and assayed for protein content for each brain region. Sample, sample + inhibitor (H-89), blank (kinase buffer and water), and pooled wells were run in duplicate and read at 450 nm and 540 nm using the BioTek Cytation^TM^ 5. The 540 nm values were subtracted from the 450 nm values as background noise. The absolute value of the difference between two absorbance readings for each sample was considered. For subjects that had technical variability in absorbance readings between duplicate values, defined as a difference in absorbance reading greater than one, the lower reading was removed. Any subject with a final negative activity value was also removed. The relative activity of PKA was quantified as the ratio of the active PKA content to the total protein content in the pooled sample for each brain region. The raw and adjusted absorbance readings as well as final PKA activity values are reported in Supplementary Table S5 and Supplementary Table S6, respectively.

### 2.3. Data Analysis

The data were analyzed using GraphPad Prism 10.0.2 (GraphPad Software Inc., La Jolla, CA, USA) and Statistica 13.0 (Statsoft Inc., Tulsa, OK, USA). The sample and inhibitor concentration data were log-transformed. Dependent measures were tested for normal distribution (D’Agostino and Pearson omnibus normality test) and homogeneity of variance (F-test). Final activity values were calculated and normalized according to the manufacturer’s protocol. Correlation analyses were performed to probe for associations between the expression of our dependent measure (PKA Activity) and subject age, tissue pH, and tissue PMI in the ACC and DLPFC. Analysis of covariance (ANCOVA) was performed if significant associations were found. If no associations were present, the data were analyzed using an unpaired two-tailed Student’s *t*-test. Slopes of the best fit lines for PKA activity were analyzed using regression analyses. α = 0.05 for all statistical tests.

## 3. Results

In this study, PKA activity in the ACC and DLPFC from schizophrenia and matched non-psychiatrically ill comparison subjects was assayed.

### 3.1. Protein and Inhibitor Concentration Optimization

**Figure 1:**
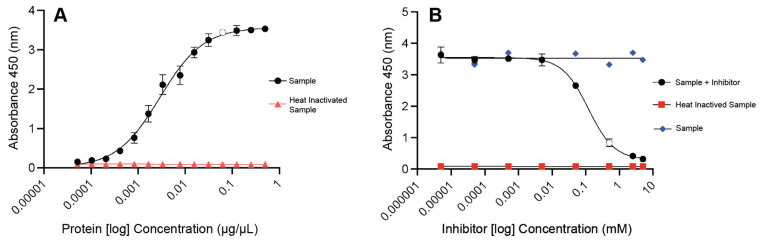
Optimization of sample and inhibitor for protein kinase A (PKA) activity assay. (**A**) Optimization of protein (sample) concentration for PKA activity assay. Optimal concentration of 0.0625 μg/μL indicated by open circle. (**B**) Optimization of inhibitor (H-89) concentration for PKA activity assay. Optimal inhibitor concentration of 0.5 mM indicated by open circle.

The optimal protein concentration (0.0625 μg/μL) (Figure 1A) and associated protein amount (1.875 μg) (Supplementary Table S3) was determined following serial dilution to ensure a linear dynamic range of protein concentrations for the PKA activity assay. The optimal concentration of the potent H-89 PKA inhibitor was determined as 0.5 mM, a concentration at which H-89 achieved a near complete inhibition of PKA (0.0625 μg/μL protein), similar to the 95 °C heat inactivation of the protein sample (Figure 1B). The 450 nm absorbance readings (without correction) of the PKA activity in the schizophrenia and control groups as well as the effect of H-89 treatment on PKA activity in these groups in the ACC and DLPFC are reported in Supplementary Figure S1. 

### 3.2. PKA Activity Assays and Correlation Analyses in the ACC and DLPFC 

**Figure 2:**
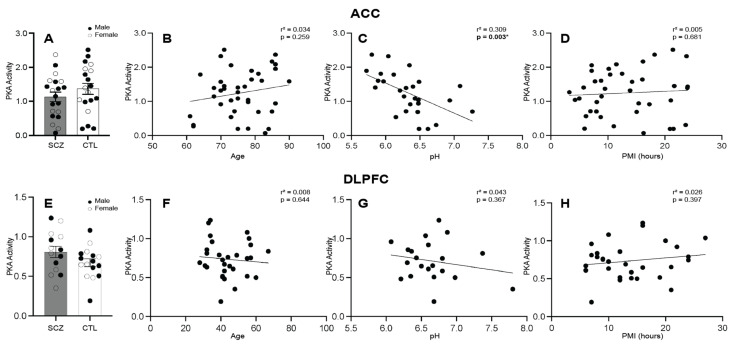
Protein kinase A (PKA) activity and correlation analyses in the anterior cingulate cortex (ACC) and dorsolateral prefrontal cortex (DLPFC) in schizophrenia (SCZ) and control (CTL) subjects. (**A**) PKA activity in SCZ (*n* = 20) and CTL (*n* = 19) groups was not significantly different in the ACC. (**B**) Correlation of PKA activity and age in SCZ and CTL subjects was not significant in the ACC. (**C**) Correlation of PKA activity and pH in SCZ and CTL subjects was significant in the ACC. (**D**) Correlation of PKA activity and PMI (hours) in SCZ and CTL subjects was not significant in the DLPFC. (**E**) PKA activity in SCZ (*n* = 14) and CTL (*n* = 16) groups was not significantly different in the DLPFC. (**F**–**H**) Correlation of PKA activity and age, PH, and PMI in SCZ and CTL subjects was not significant in the DLPFC. Data presented as mean ± standard error of the mean (SEM). * *p* < 0.05.

The optimized PKA activity assay was performed with ACC and DLPFC tissue homogenate samples. The PKA activity was not significantly different between the schizophrenia and control groups in the ACC (ANCOVA: F_(1,24)_ = 2.680, *p* = 0.115, after controlling for the effect of pH, Figure 2A) or in the DLPFC (t_(28)_ = 1.598, *p* = 0.121, Figure 2E). 

The correlation analyses showed no significant associations in the schizophrenia and control groups between PKA activity and age (Spearman’s r = 0.034, *p* = 0.259) or PMI (Spearman’s r = 0.005, *p* = 0.681) in the ACC; however, a significant association was found between PKA activity and pH (Spearman’s r = 0.309, *p* = 0.003) in the ACC (Figure 2B–D). The correlation analyses showed no significant associations in the schizophrenia and control groups between PKA activity and age (Spearman’s r = 0.008, *p* = 0.664), pH (Spearman’s r = 0.043, *p* = 0.367), or PMI (Spearman’s r = 0.026, *p* = 0.397) in the DLPFC (Figure 2F–H). 

In the ACC, PKA activity was not significantly different between the male schizophrenia and male control groups (t_(21)_ = 0.714, *p* = 0.483, Supplementary Figure S2A). PKA activity was also not significantly different between the female schizophrenia and female control groups (ANCOVA: F_(1,10)_ = 2.822, *p* = 0.124, after controlling for the effect of pH, Supplementary Figure S2E). In the DLPFC, PKA activity was not significantly different between the male schizophrenia and male control groups (t_(13)_ = 1.272, *p* = 0.226, Supplementary Figure S3A) or female schizophrenia and female control groups (t_(13)_ = 0.909, *p* = 0.380, Supplementary Figure S3E). 

Correlation plots are presented showing associations between PKA activity and age, pH, and PMI in the ACC in male subjects (Supplementary Figure S2B–D) and female subjects (Supplementary Figure S2F–H) as well as in the DLPFC in male subjects (Supplementary Figure S3B–D) and female subjects (Supplementary Figure S3F–H). In the ACC, there was a significant association in the female subjects between PKA activity and age (Spearman’s r = 0.311, *p* = 0.025) and pH (Spearman’s r = 0.632, *p* = 0.001), but not PMI. In the DLPFC, no significant associations were found in either the male or female groups.

### 3.3. Antipsychotic Effects on PKA Activity 

PKA activity was analyzed in schizophrenia subjects who were “on” compared to “off” medication at time of death. “Off” medication indicates subjects who were off antipsychotics for at least 6 weeks prior to death. Subjects whose medication status was unknown were excluded from the analysis. PKA activity was not significantly impacted by medication status in the ACC (*p* > 0.05). This analysis was not performed for schizophrenia subjects in the DLPFC, as not enough subjects were off antipsychotic medication at time of death to perform a meaningful statistical comparison.

## 4. Discussion

The dysregulation of several kinase-mediated signaling events is implicated in the pathophysiology of schizophrenia [29,49]; however, the functional changes in PKA in the brain in this disorder are still poorly understood, with few studies examining the activity of this enzyme. Here, we assayed PKA activity in the ACC and DLPFC to determine whether the enzyme activity was significantly altered in subjects with schizophrenia. Contrary to our initial hypothesis, we did not observe significant differences in PKA activity between the schizophrenia and non-psychiatrically ill control subjects in either the ACC or DLPFC. We also did not observe significant sex differences in PKA activity in either brain region. Our previous studies examining dysregulated kinase-mediated events in the brain in schizophrenia implicated changes in PKA activity in the ACC [31,32]. The inability to replicate this finding may be due to differences in the methods applied, as the former studies utilized an array-based technology for analyzing several active kinases [50], while the present study used a specific kinase activity assay. 

To accurately interpret our findings, the underlying mechanism of PKA activation in the brain must be considered. PKA is a key downstream signaling molecule of the G-protein-coupled receptor (GPCR) pathway and may be activated by both neurotransmitter-controlled and neuropeptide-controlled GPCRs [51,52]. Heterotrimeric G-proteins are composed of the Gα, Gβ, and Gγ subunits, and Gα subunits are further classified into four categories, Gαs, Gαi, Gαq, and Gα_12–13_, all of which modulate adenylyl cyclase, cAMP, and protein kinases implicated in schizophrenia such as PKA, protein kinase C (PKC), and protein kinase G (PKG) to various degrees [53,54,55]. Although these are distinct families of protein kinases that typically function independently, instances of crosstalk and interactions between these signaling pathways often occur [49]. The overall mechanisms underlying changes in PKA activity in schizophrenia are therefore extremely complex. While our study did not find changes in PKA activity between schizophrenia and control subjects in the ACC or DLPFC, significant changes may exist in PKA mRNA and/or protein levels. Additionally, significant changes may exist in the mRNA expression, protein levels, and/or activity of other protein kinases such as PKC or PKG in human postmortem brain tissue between schizophrenia and control subjects and should be further explored to gain a broader understanding of kinase signaling perturbations in this disorder.

The factors leading to changes in PKA activity in schizophrenia are also multifactorial. The overactivity of the dopamine D_2_ receptors, which are also GPCRs, contributes to the positive symptoms observed in schizophrenia and is correlated with PKA dysregulation [56]. Alterd glutamatergic signaling, which leads to the dysregulation of NMDA and AMPA receptors, also affects PKA activity [27,57]. The dysfunction of neuropeptide signaling pathways acting through GPCRs, such as neuropeptide Y or substance P, has also been implicated in schizophrenia and PKA signaling abnormalities [58]. Additionally, the antipsychotic medications commonly used to treat schizophrenia alter the balance of neurotransmitters, GPCR signaling, and PKA function [59]. Our work contributes to the growing body of literature assessing the mechanisms and implications of altered PKA activity in this illness by exploring some of these factors.

Previous studies investigating the protein abundance of the catalytic form of PKA found no significant changes in protein levels in the DLPFC in schizophrenia [49], although this study, and others [60], identified changes in other cAMP-related signaling molecules. Interestingly, increased phospho-PKA levels were reported in endothelial cells in the prefrontal cortex, but not the visual cortex, in schizophrenia subjects [61]. This suggests that alterations in PKA activity in schizophrenia may be brain-region- and cell-subtype-specific. Since we assessed PKA activity at the region level, our results are a measure of enzyme activity in all cells in the tissues and may not capture activity changes in specific cell types [31,62], as the increased activity in one cell type may be negated by the decreased activity in another cell type. Furthermore, underlying changes in the structure of brain tissue [63] and the morphology of specific cell types, such as neurons [64], between schizophrenia and control subjects may impact communication between cells and PKA activity; however, our study did not detect a change in PKA activity between the schizophrenia and control subjects in the ACC or DLPFC. One plausible explanation is that that these nuances may have been masked at the region level. Future studies exploring different cell subtypes in the frontal cortex, or other implicated brain regions, may provide additional insight into changes in PKA or other protein kinase activity in schizophrenia. 

Another important consideration is the selection of brain regions in our study. Although we assessed changes in PKA activity in the ACC and DLPFC due to their structural and functional involvement in schizophrenia pathology, other regions of the brain are also implicated in this disorder such as the temporal lobe, hippocampus, and striatum [65,66,67]. Tissue from these brain regions was not readily available for analysis in our study; however, PKA activity in these brain regions may be significantly altered, and future studies should focus on understanding these variations to gain a more robust insight into PKA function in schizophrenia. Furthermore, while our study focused on PKA function via activity assays, PKA may also be altered at the mRNA and/or protein level, and these changes may not be correlated with each other or with activity changes. Therefore, although we did not observe PKA activity changes in the ACC or DLPFC, significant changes in PKA mRNA and/or protein levels are possible and should be considered not only in the frontal cortex, but also in any brain region implicated in schizophrenia, utilizing RT-qPCR and Western blot techniques in future studies.

The potential confounding variables on PKA enzyme activity include age, tissue pH, and PMI, as they influence the molecular and biochemical properties of tissue; however, studies have reported the relative stability of enzyme activity in postmortem tissue [68,69], providing further support for measuring PKA activity in postmortem tissue. Still, we accounted for these variables via covariate analyses for each comparison group of interest. In the DLPFC, the enzyme activity findings did not appear to be due to an age, pH, or PMI effect. In the ACC, PKA activity was significantly correlated with pH in all subjects and with both age and pH in female subjects. After controlling for these variables, we did not observe significant differences in PKA activity in either group; however, we cannot exclude the possibility that these variables may have had confounding effects on the assay. The significant correlations found in our analysis support the broader notion of sexual dimorphism that has been well documented in the presentation and course of schizophrenia [70] and warrant future studies exploring the impact of metabolic factors on PKA activity or studies focused on PKA activity across various neurodevelopmental stages in both sexes to better understand the effects of pH and age on PKA activity. One limitation of this analysis is that it may not accurately detect the nuances of how covariates affect specific cell populations. The complexity of the tissue (e.g., different cell types and tissue layers) may have varying responses to covariates. Techniques that may provide such specificity have not yet been adapted to postmortem tissue [71].

An additional variable that may impact PKA enzyme activity is chronic physiological stress. Several studies have demonstrated elevated cortisol levels in subjects with schizophrenia, typically due to lower socioeconomic status, higher rates of drug use, and greater cognitive impairment compared to healthy control subjects [72,73,74]. A limitation of our study is that assays have not yet been adapted to measure cortisol levels using brain homogenate. Future studies may focus on developing such assays or utilizing alternative statistical techniques to examine the correlations between other stress-related biomarkers in schizophrenia, such as brain-derived neurotrophic factor (BDNF) and PKA activity [75], depending on the availability of this information from the brain bank(s) utilized.

We also studied the effect of antipsychotic treatment on PKA activity and found no significant changes in activity due to medication. This analysis was only conducted in one brain region, the ACC, due to the relatively small number of subjects who were “off” medication in the DLPFC cohort, which is not surprising given the severity of this disorder. An important consideration of this analysis is that information regarding the use of over the counter (OTC) medications (e.g., medication type and duration of use) by all subjects prior to death may have had an impact on PKA activity; however, information regarding OTC medication prescriptions were not readily available to us for use in this study. Further studies with larger subject numbers and consideration of non-antipsychotic medications, when possible, are warranted to determine the potential effects of antipsychotics on PKA activity more accurately. 

PKA is a ubiquitously expressed protein kinase with dynamic changes in activity. Cell- and compartment-specific patterns of protein kinase expression exist in the brain [76]. A limitation of this study is that we used blended brain homogenate samples for our assays; however, this was carried out to ensure representative sampling across different regions of the brain to provide a comprehensive overview of molecular tissue characteristics. An inherent limitation of postmortem studies is that the primary endpoint is death. Thus, when considering changes in activity over time, we are limited to only assessing the correlations between age at death and our dependent measures. To gain an understanding of PKA activity in schizophrenia across several time points in postmortem studies using our assay, investigators should aim to collect and analyze tissue across various age groups.

Although the present assay is a gold-standard confirmatory technique employed to assess whether we could confirm our previous finding of changes in PKA activity in schizophrenia and has been validated for accurately detecting PKA activity levels across several research domains including neurotherapeutics, cardiovascular, and cancer [77,78,79], we spent considerable time optimizing the assay. We optimized the concentration of the potent H-89 PKA inhibitor to demonstrate the specificity of our assay and included appropriate positive and negative controls. Overall, we performed every experiment following best practices for rigor and reproducibility to minimize the impact of false results.

Lastly, our study utilized cohorts of approximately 15–20 subjects. The sex-specific and antipsychotic medication analyses used fewer subjects in each brain region. In the present study, we did not detect a difference between the schizophrenia and control subject groups. We are not asserting that there is, in fact, no difference, only that we did not detect a difference. Future studies should incorporate larger sample sizes when possible, to enhance the generalizability of the insights provided by this study on altered PKA activity in the frontal cortex in schizophrenia.

## 5. Conclusions

PKA is implicated in the pathophysiology of neuropsychiatric disorders [80,81,82], but few studies have explored PKA activity in the brain in schizophrenia. This study is the first to investigate PKA enzyme activity in the DLPFC and ACC in subjects diagnosed with this disorder. Our findings highlight the need for future cell-subtype, sex-specific studies to continue to explore altered kinase networks in schizophrenia. Postmortem studies should focus on isolating neurons, as PKA is the most abundant in these cell types [83] from the ACC and DLPFC, as well as other brain regions highly implicated in this disorder, such as the hippocampus and striatum. Given that sexual dimorphism has been well documented in the presentation and course of schizophrenia, future research with larger sample sizes should continue to explore sex-specific alterations in PKA activity, considering the effects of potential confounding variables such as age, tissue pH, PMI, chronic physiological stress, and OTC medications. Lastly, this work should be expanded upon to investigate the mRNA expression, protein abundance, and activity of other kinase targets in schizophrenia, as they may impact intracellular calcium levels, neurotransmitter receptors, crosstalk between signaling pathways, and other biological functions critical for neuroplasticity, ultimately building upon the insights provided by our investigation. 

## Figures and Tables

**Table 1:** Demographics of anterior cingulate cortex (ACC) and dorsolateral prefrontal cortex (DLPFC) subjects used in the study. Data presented as mean ± standard deviation. Data range in parentheses. Abbreviations: N, number of subjects; F, female; M, male; PMI, postmortem interval; UNK, unknown; N/A, not applicable.

	N	Sex	Age	PMI (hours)	Medication Status	pH
Schizophrenia (ACC)	20	9F/11M	75 ± 8 (61–90)	13.1 ± 5.8 (5.8–24)	12 ON/6 OFF/2 UNK	6.3 ± 0.2 (5.8–6.7)
Control (ACC)	19	7F/12M	76 ± 7 (62–86)	13.2 ± 7.1 (3.3–24)	N/A	6.5 ± 0.5 (5.7–7.3)
Schizophrenia (DLPFC)	15	8F/7M	45 ± 11 (32–67)	14.9 ± 6.3 (6–27)	9 ON/1 OFF/5 UNK	6.6 ± 0.5 (6.1–7.8)
Control (DLPFC)	18	9F/9M	42 ± 11 (23–60)	12.6 ± 5.3 (6–24)	N/A	6.7 ± 0.3 (6.2–7.4)

## Data Availability

Data are contained within the article and Supplementary Materials.

## Supplementary Materials

The following supporting information can be downloaded at: https://www.mdpi.com/article/10.3390/brainsci14010013/s1, Supplementary Methods: Rationale for studying the ACC and DLPFC; Table S1: Demographics of anterior cingulate cortex (ACC) subjects from the Mount Sinai NIH Brain and Tissue Repository used in study. Table S2: Demographics of dorsolateral prefrontal cortex (DLPFC) subjects from the Maryland Brain Collection used in study. Table S3: Protein concentrations, associated amounts, and associated raw absorbance values. Table S4: Inhibitor (H-89) concentration and associated raw absorbance values. Table S5: Raw and adjusted absorbance readings as well as final activity from protein kinase A (PKA) activity assay for anterior cingulate cortex (ACC) subjects. Table S6: Raw and adjusted absorbance readings as well as final activity from protein kinase A (PKA) activity assay for dorsolateral prefrontal cortex (DLPFC) subjects. Figure S1: Protein kinase A (PKA) activity in the anterior cingulate cortex (ACC) and dorsolateral prefrontal cortex (DLPFC) in schizophrenia (SCZ) and control (CTL) subjects with inhibitor (H-89) and vehicle (blank). Figure S2: Protein kinase A (PKA) activity and correlation analyses in the anterior cingulate cortex (ACC) in schizophrenia (SCZ) and control (CTL) subjects based on sex. Figure S3: Protein kinase A (PKA) activity and correlation analyses in the dorsolateral prefrontal cortex (DLPFC) in schizophrenia (SCZ) and control (CTL) subjects based on sex. Supplementary References.
