# Supplementary material for: Activity of Protein Kinase A in the Frontal Cortex in Schizophrenia
Source: Brain Sci. 2023 Dec 22;14(1):13. doi: 10.3390/brainsci14010013 (PMC10813263; doi:10.3390/brainsci14010013)
Supplement: Supplementary file 1 [file brainsci-14-00013-s001.zip › brainsci-2668946-supplementary.pdf]

Supplementary Material.

Contents.

1. **Supplementary Methods** Page 2
2. **Supplementary Tables & Table Captions** Page 3
3. **Supplementary Figures & Captions** Page 9
4. **Supplementary References** Page 12

Supplementary Methods.

Rationale for studying the ACC and DLPFC. Among the cortical areas that are functionally and/or structurally affected in schizophrenia, the anterior cingulate cortex (ACC) and dorsolateral prefrontal cortex (DLPFC) are commonly involved.

The ACC consistently exhibits abnormalities linked to cognitive dysfunction and disrupted connectivity, leading to disordered thought processes and difficulty adapting to changing situations [1]. Additionally, the ACC contains a high density of dopamine receptors and is sensitive to changes in dopamine levels [2]. Functional neuroimaging studies have consistently shown altered ACC activity in individuals with schizophrenia during cognitive and emotional tasks, providing support for this region's involvement in this disorder [3, 4].

The DLPFC is involved in executive functioning and top-down regulation of goal-oriented behavior [5]. In schizophrenia, the DLPFC is altered in working memory tasks, which are hypothesized to be impaired several years prior to diagnosis [6]. Additionally, grey matter volume is reduced in this brain region due to reduced neuropil in schizophrenia [7]. The DLPFC is interconnected with subcortical regions, including the striatum, and dysregulation of prefrontal-striatal circuitry is implicated in higher order processes and behavioral pathology in schizophrenia [8].

Taken together, understanding the activity of PKA in these brain regions may help decipher the intricate alterations that underlie the hypothesized dopaminergic and glutamatergic abnormalities in this illness.

Supplementary Tables & Table Captions.

SUBJECT #	AGE	RACE	SEX	PH	PMI	MED STATUS	DIAG
1	73	W	M	6.15	8.8	ON	Schizophrenia
2	77	W	M	6.4	24.0	OFF	Schizophrenia
3	73	W	M	6.35	7.2	ON	Schizophrenia
4	70	W	M	6.49	14.3	ON	Schizophrenia
5	68	W	M	6.27	8.9	ON	Schizophrenia
6	86	W	M	6.48	15.4	ON	Schizophrenia
7	75	W	M	5.85	5.8	ON	Schizophrenia
8	70	W	M	6.36	17.3	OFF	Schizophrenia
9	71	W	M	N/A	9.5	ON	Schizophrenia
10	83	W	M	N/A	16.3	OFF	Schizophrenia
11	61	W	M	N/A	6.2	ON	Schizophrenia
12	70	B	F	6.21	13.9	UNK	Schizophrenia
13	74	W	F	6.3	7.0	ON	Schizophrenia
14	81	W	F	5.93	12.5	OFF	Schizophrenia
15	82	W	F	5.89	8.8	OFF	Schizophrenia
16	90	W	F	5.97	7.8	ON	Schizophrenia
17	81	W	F	6.47	15.1	OFF	Schizophrenia
18	62	W	F	6.74	23.7	ON	Schizophrenia
19	75	W	F	6.49	21.5	UNK	Schizophrenia
20	86	W	F	5.8	18.2	ON	Schizophrenia
21	64	W	M	6.12	10.4	N/A	Control
22	70	W	M	6.04	23.8	N/A	Control
23	78	W	M	N/A	8.1	N/A	Control
24	71	W	M	N/A	21.4	N/A	Control
25	62	W	M	N/A	11.1	N/A	Control
26	85	N/A	M	N/A	16.2	N/A	Control
27	75	B	M	6.43	5.0	N/A	Control
28	71	W	M	7.09	21.7	N/A	Control
29	79	W	M	N/A	16.1	N/A	Control
30	84	W	M	6.62	20.9	N/A	Control
31	78	W	M	N/A	5.9	N/A	Control
32	86	W	F	N/A	10.2	N/A	Control
33	75	H	F	N/A	3.3	N/A	Control
34	85	W	F	7.27	8.0	N/A	Control
35	79	W	F	5.72	7.2	N/A	Control
36	78	W	F	6.48	4.3	N/A	Control
37	86	W	F	N/A	11.5	N/A	Control
38	68	W	F	6.30	24.0	N/A	Control
39	73	W	M	6.94	21.1	N/A	Control

Supplementary Table S1 (S1). Demographics of anterior cingulate cortex (ACC) subjects from the Mount Sinai NIH Brain and Tissue Repository used in study. Subject identification numbers, age, race, sex, pH, PMI, medication status, and diagnosis listed for all ACC subjects used in study. *Abbreviations*: *PMI*, postmortem interval (hours); *MED*, medication; *UNK*, unknown; *DIAG*, diagnosis; *W*, white; *B*, black; *F*, female; *M* male; *N/A*, not available.

SUBJECT #	AGE	RACE	SEX	PH	PMI	MED STATUS	DIAG
1	55	W	M	6.35	12	ON	Schizophrenia
2	56	W	F	N/A	20	UNK	Schizophrenia
3	42	W	M	N/A	6	ON	Schizophrenia
4	42	W	F	6.8	14	ON	Schizophrenia
5	35	W	M	6.07	7	ON	Schizophrenia
6	32	B	F	6.31	12	ON	Schizophrenia
7	34	W	M	6.75	16	ON	Schizophrenia
8	33	W	F	N/A	16	UNK	Schizophrenia
9	54	W	M	6.61	12	OFF	Schizophrenia
10	34	W	F	6.56	27	UNK	Schizophrenia
11	48	W	F	7.8	21	UNK	Schizophrenia
12	67	B	M	6.36	8	ON	Schizophrenia
13	39	W	F	N/A	24	UNK	Schizophrenia
14	57	B	M	6.43	9	ON	Schizophrenia
15	41	B	F	N/A	19	ON	Schizophrenia
16	55	W	M	6.87	10	N/A	Control
17	57	W	F	6.6	22	N/A	Control
18	47	W	M	6.6	6	N/A	Control
19	42	W	F	6.21	12	N/A	Control
20	32	W	M	N/A	10	N/A	Control
21	37	W	F	6.49	10	N/A	Control
22	40	B	M	6.68	7	N/A	Control
23	32	W	F	7.37	7	N/A	Control
24	28	W	M	6.3	13	N/A	Control
25	23	W	F	6.54	12	N/A	Control
26	49	W	M	N/A	8	N/A	Control
27	31	W	F	6.66	21	N/A	Control
28	41	W	M	N/A	10	N/A	Control
29	60	W	F	6.98	15	N/A	Control
30	44	B	M	N/A	9	N/A	Control
31	55	B	F	6.8	24	N/A	Control
32	41	B	M	6.68	14	N/A	Control
33	45	W	F	6.50	16	N/A	Control

Supplementary Table S2 (S2). Demographics of dorsolateral prefrontal cortex (DLPFC) subjects from the Maryland Brain Collection used in study. Subject identification numbers, age, race, sex, pH, PMI, medication status, and diagnosis listed for all DLPFC subjects used in study. *Abbreviations*: *PMI,* postmortem interval (hours); *MED*, medication; *UNK*, unknown *DIAG*, diagnosis; *W*, white; *B*, black; *F,* female; *M* male; *N/A*, not available.

Protein Concentration (µg/µL)	Protein (µg)	Sample Absorbances (450 nm) (in triplicate)			Heat Inactivated Sample Absorbances (450 nm) (in triplicate)		
0.5	15	3.623	3.499	3.477	0.083	0.084	0.095
0.25	7.5	3.555	3.489	3.458	0.089	0.082	0.078
0.125	3.75	3.619	3.488	3.358	0.09	0.097	0.083
**0.0625**	**1.875**	**3.488**	**3.43**	**3.404**	0.083	0.085	0.094
0.03125	0.9375	3.383	3.279	3.069	0.091	0.097	0.086
0.015625	0.46875	3.053	2.961	2.788	0.087	0.088	0.085
0.0078	0.234375	2.501	2.474	2.084	0.084	0.087	0.107
0.00333	0.1171875	2.4	1.942	1.992	0.096	0.092	0.098
0.001667	0.05859375	1.55	1.434	1.138	0.09	0.087	0.098
0.0008325	0.02929688	0.891	0.779	0.625	0.094	0.093	0.094
0.00041625	0.01464844	0.455	0.441	0.403	0.104	0.093	0.093
0.000208125	0.00732422	0.263	0.239	0.183	0.094	0.09	0.088
0.000104063	0.00366211	0.2	0.192	0.177	0.096	0.093	0.094
5.20313E-05	0.00183105	0.153	0.151	0.148	0.097	0.092	0.095

Supplementary Table S3 (S3). **Protein concentrations, associated amounts, and associated raw absorbance values**. All sample and heat inactivated samples were run in triplicate for each concentration. Bold numbers indicate the optimal protein concentration in µg/µL, protein amount in µg, and associated absorbance values for the samples.

**Inhibitor Concentration (mM)**	**Sample**	**Sample + Inhibitor Absorbances (450 nm) (in duplicate)**		**Heat inactivated Sample Absorbances (450 nm)**
5	3.471	0.287	0.349	0.08
2.5	3.696	0.42	0.412	0.082
**0.5**	3.322	**0.92**	**0.745**	0.087
0.05	3.672	2.648	2.663	0.083
0.005	3.471	3.336	3.61	0.08
0.0005	3.696	3.505	3.513	0.082
0.00005	3.322	3.399	3.551	0.087
0.000005	3.672	3.443	3.807	0.083

Supplementary Table S4 (S4). **Inhibitor (H-89) concentration and associated raw absorbance values**. The sample + inhibitor was run in duplicate for each inhibitor concentration. Bold numbers indicate the optimal inhibitor concentration in mM and associated absorbance values for the sample + inhibitor.

Diagnosis	Sample Absorbances (450 nm)	Sample + Inhibitor Absorbances (450 nm)	Adjusted Absorbances	Final Normalized Activity
M SCZ	1.134	0.889	0.245	0.542
M SCZ	1.137	0.489	0.648	1.434
F SCZ	1.164	0.438	0.726	1.607
M CTL	1.262	0.4555	0.807	1.785
M CTL	1.311	0.82	0.491	1.086
M SCZ	1.1895	0.6715	0.518	1.146
F SCZ	1.3465	0.751	0.596	1.318
M CTL	1.405	0.749	0.656	1.452
M CTL	1.437	0.3875	1.050	2.323
M SCZ	1.477	0.7665	0.711	1.573
F CTL	1.626	0.7435	0.883	1.953
F CTL	1.345	0.7155	0.630	1.393
F CTL	1.289	0.433	0.856	1.895
M SCZ	1.49	0.5605	0.930	2.058
M CTL	1.5245	0.5445	0.980	2.169
M CTL	1.5935	0.458	1.136	2.514
M CTL	1.2215	0.4795	0.742	1.642
F SCZ	1.0175	0.199	0.819	1.812
F CTL	1.4755	1.157	0.319	0.704
F CTL	1.445	0.5025	0.943	2.086
F SCZ	1.5335	0.464	1.070	2.368
F SCZ	0.962	0.5255	0.437	0.690
F SCZ	0.9495	0.825	0.125	0.197
F CTL	1.248	0.446	0.802	1.268
M SCZ	1.2035	1.1585	0.045	0.071
F SCZ	1.405	0.4015	1.004	1.587
M CTL	1.312	1.189	0.123	0.194
M SCZ	1.543	0.6545	0.889	1.405
M SCZ	1.355	0.7755	0.580	0.916
M CTL	1.396	1.2685	0.128	0.202
M SCZ	1.5165	0.921	0.596	0.942
F CTL	1.451	0.7915	0.660	1.043
M CTL	1.612	0.9915	0.621	0.981
M SCZ	1.489	0.622	0.867	1.371
M SCZ	1.4405	1.082	0.359	0.567
M CTL	1.504	1.333	0.171	0.270
F SCZ	1.4855	1.036	0.450	0.711
F SCZ	1.472	1.2775	0.195	0.308
M CTL	1.5515	0.9015	0.650	1.028

Supplementary Table S5 (S5). Raw and adjusted absorbance readings as well as final activity from protein kinase A (PKA) activity assay for anterior cingulate cortex (ACC) subjects. The raw absorbances for each sample and sample + inhibitor as well as adjusted absorbances and normalized PKA activity values are presented for all subjects used in the study. *Abbreviations*: *SCZ,* schizophrenia; *CTL,* control; *F,* female; *M* male.

Diagnosis	Sample Absorbances (450 nm)	Sample + Inhibitor Absorbances (450 nm)	Adjusted Absorbances	Final Normalized Activity
M CTL	1.691	0.3785	1.313	1.081
M CTL	1.6685	0.846	0.823	0.609
M CTL	2.0495	1.2025	0.847	0.633
M CTL	1.201	0.815	0.386	0.190
M CTL	1.528	0.622	0.906	0.690
M CTL	1.828	0.8215	1.007	0.786
F SCZ	1.609	0.3805	1.229	1.000
F SCZ	1.5545	0.7575	0.797	0.585
F SCZ	1.42	0.368	1.080	0.857
F SCZ	1.3825	0.6585	0.724	0.515
F SCZ	1.612	0.1745	1.438	1.201
F SCZ	1.846	0.5795	1.267	1.037
F CTL	1.616	0.4715	1.145	0.919
F CTL	1.4605	0.7705	0.690	0.482
F CTL	1.613	0.5825	1.031	0.810
M SCZ	0.978	0.2535	0.725	0.515
M SCZ	1.5645	0.6805	0.884	0.669
M SCZ	1.552	0.366	1.186	0.959
M SCZ	1.515	0.543	0.972	0.753
M SCZ	1.724	0.2515	1.473	1.235
F CTL	1.3725	0.505	0.868	0.653
F CTL	1.154	0.4465	0.708	0.499
F CTL	1.509	0.545	0.964	0.746
M CTL	1.3165	0.6015	0.715	0.506
M SCZ	1.47	0.4125	1.058	0.835
M CTL	1.563	0.619	0.944	0.726
M CTL	1.4705	0.49	0.981	0.761
F CTL	1.276	0.414	0.862	0.647
F SCZ	0.775	0.222	0.553	0.350
F SCZ	1.5925	0.5835	1.009	0.789

Supplementary Table S6 (S6). Raw and adjusted absorbance readings as well as final activity from protein kinase A (PKA) activity assay for dorsolateral prefrontal cortex (DLPFC) subjects. The raw absorbances for each sample and sample + inhibitor as well as adjusted absorbances and normalized PKA activity values are presented for all subjects used in the study. *Abbreviations*: *SCZ,* schizophrenia; *CTL*, control; *F,* female; *M* male.

Supplementary Figures & Figure Captions.

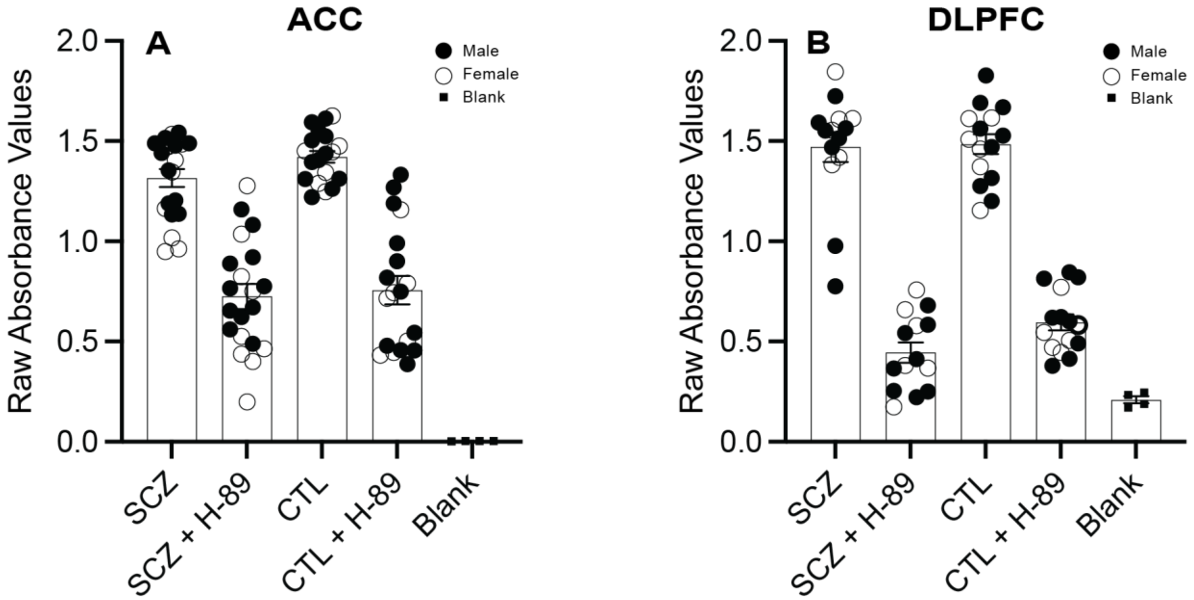

Supplementary Figure S1 (S1). **Protein kinase A (PKA) activity in the anterior cingulate cortex (ACC) and dorsolateral prefrontal cortex (DLPFC) in schizophrenia (SCZ) and control (CTL) subjects with inhibitor (H-89) and vehicle (blank)**. A) Raw absorbance values are significantly different in the following groups in the ACC: SCZ and SCZ + H-89, SCZ and Blank, CTL and CTL + H-89, and CTL and Blank. B) Raw absorbance values are significantly different in the following groups in the DLPFC: SCZ and SCZ + H-89, SCZ and Blank, CTL and CTL + H-89, and CTL and Blank. Pairwise comparisons not shown. $p < 0.0001$.

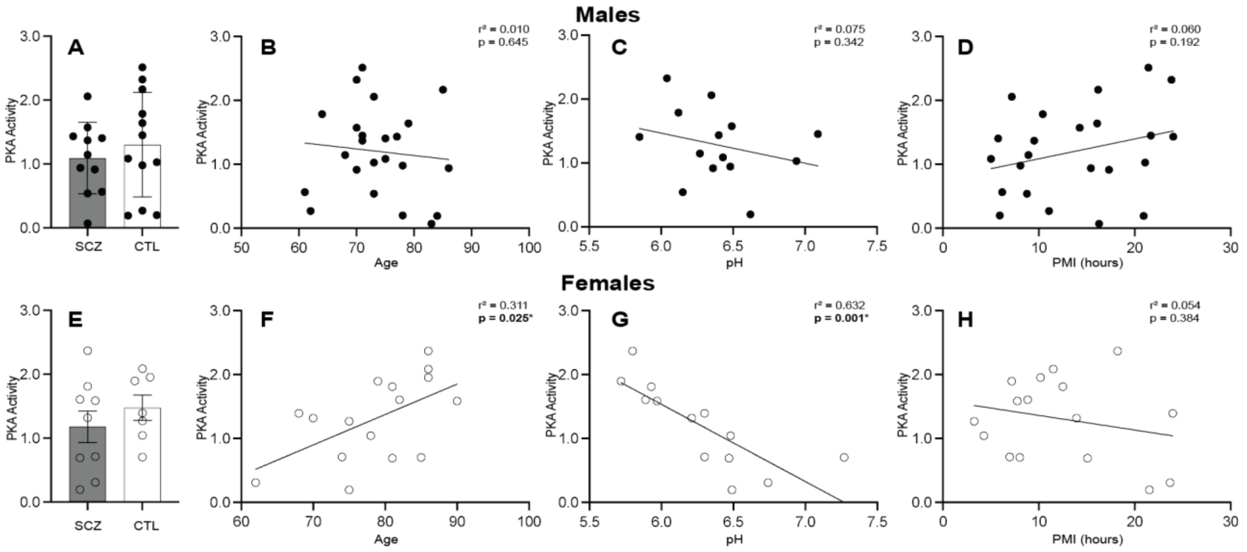

Supplementary Figure S2 (S2). **Protein kinase A (PKA) activity and correlation analyses in the anterior cingulate cortex (ACC) in male and female schizophrenia (SCZ) and control (CTL) subjects**. A) PKA activity in SCZ male (n = 11) and CTL male (n = 12) groups was not significantly different in the ACC. B-D) Correlation of PKA activity and age, PH, and PMI in SCZ and CTL male subjects was not significantly different in the ACC. E) PKA activity in SCZ female subjects (n = 9) and CTL female subjects (n = 7) groups was not significantly different in the ACC. F) Correlation of PKA activity and age in SCZ and CTL female subjects was significant in the ACC. G) Correlation of PKA activity and pH in SCZ and CTL female subjects was significant in the ACC. H) Correlation of PKA activity and PMI (hours) in SCZ and CTL female subjects was not significant in the ACC. Data presented as mean ± standard error of the mean (SEM). * $p < 0.05$.

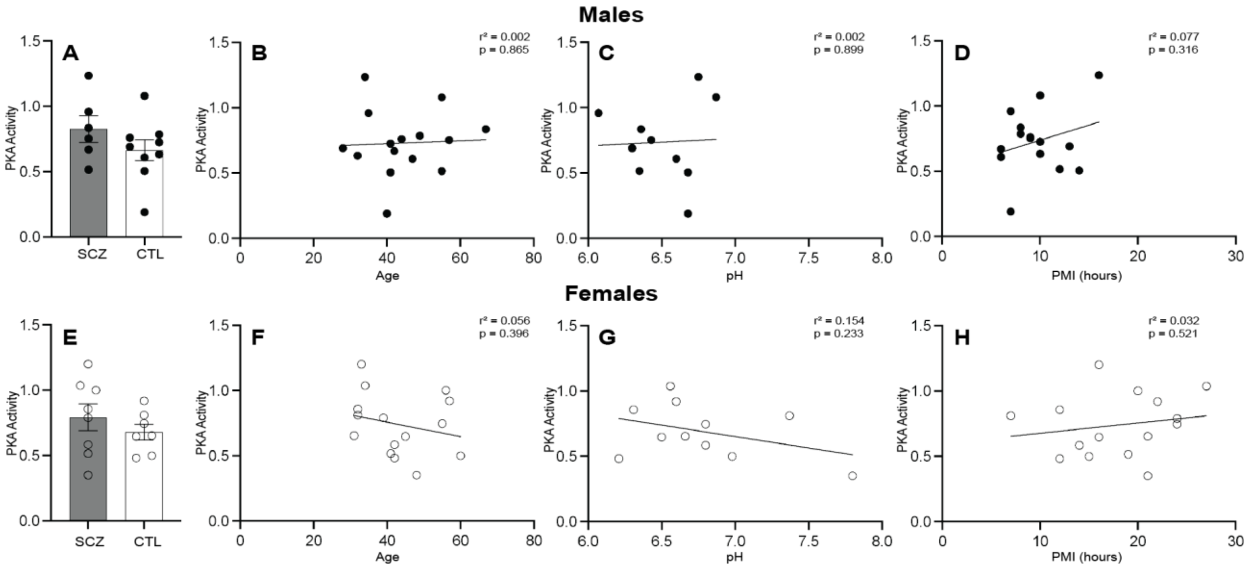

Supplementary Figure S3 (S3). **Protein kinase A (PKA) activity and correlation analyses in the dorsolateral prefrontal cortex (DLPFC) in male and female schizophrenia (SCZ) and control (CTL) subjects**. A) PKA activity in SCZ male (n = 6) and CTL male (n = 9) groups was not significantly different in the DLPFC. B-D) Correlation of PKA activity and age, PH, and PMI in SCZ and CTL male subjects was not significantly different in the DLPFC. E) PKA activity in SCZ female subjects (n = 8) and CTL female subjects (n = 6) groups was not significantly different in the DLPFC. F-H) Correlation of PKA activity and age, PH, and PMI in SCZ and CTL female subjects was not significantly different in the DLPFC. Data presented as mean ± standard error of the mean (SEM). * $p < 0.05$.

Supplementary References.

[1] J. D. Henry, W. von Hippel, P. Molenberghs, T. Lee, and P. S. Sachdev, "Clinical assessment of social cognitive function in neurological disorders," (in eng), *Nat Rev Neurol,* vol. 12, no. 1, pp. 28-39, Jan 2016, doi: 10.1038/nrneurol.2015.229.

[2] F. M. Benes, M. S. Todtenkopf, and J. B. Taylor, "Differential distribution of tyrosine hydroxylase fibers on small and large neurons in layer II of anterior cingulate cortex of schizophrenic brain," (in eng), *Synapse,* vol. 25, no. 1, pp. 80-92, Jan 1997, doi: 10.1002/(SICI)1098-2396(199701)25:1<80::AID-SYN10>3.0.CO;2-2.

[3] D. C. Glahn *et al.*, "Beyond hypofrontality: a quantitative meta-analysis of functional neuroimaging studies of working memory in schizophrenia," (in eng), *Hum Brain Mapp,* vol. 25, no. 1, pp. 60-9, May 2005, doi: 10.1002/hbm.20138.

[4] S. F. Taylor, J. Kang, I. S. Brege, I. F. Tso, A. Hosanagar, and T. D. Johnson, "Meta-analysis of functional neuroimaging studies of emotion perception and experience in schizophrenia," (in eng), *Biol Psychiatry,* vol. 71, no. 2, pp. 136-45, Jan 15 2012, doi: 10.1016/j.biopsych.2011.09.007.

[5] D. J. Furman, J. P. Hamilton, and I. H. Gotlib, "Frontostriatal functional connectivity in major depressive disorder," (in eng), *Biol Mood Anxiety Disord,* vol. 1, no. 1, p. 11, Dec 08 2011, doi: 10.1186/2045-5380-1-11.

[6] T. A. Lesh, T. A. Niendam, M. J. Minzenberg, and C. S. Carter, "Cognitive control deficits in schizophrenia: mechanisms and meaning," (in eng), *Neuropsychopharmacology,* vol. 36, no. 1, pp. 316-38, Jan 2011, doi: 10.1038/npp.2010.156.

[7] L. A. Glantz and D. A. Lewis, "Reduction of synaptophysin immunoreactivity in the prefrontal cortex of subjects with schizophrenia. Regional and diagnostic specificity," (in eng), *Arch Gen Psychiatry,* vol. 54, no. 10, pp. 943-52, Oct 1997, doi: 10.1001/archpsyc.1997.01830220065010.

[8] C. R. Sullivan, S. M. O'Donovan, R. E. McCullumsmith, and A. Ramsey, "Defects in Bioenergetic Coupling in Schizophrenia," (in eng), *Biol Psychiatry,* vol. 83, no. 9, pp. 739-750, 05 01 2018, doi: 10.1016/j.biopsych.2017.10.014.
